# Association of Lesion Topography with Functional Outcomes in Acute Ischemic Stroke Patients Considered for, or Receiving, Reperfusion Therapy: A Meta-Analysis

**DOI:** 10.3390/neurolint14040073

**Published:** 2022-11-07

**Authors:** Shuyue Chen, Kevin J. Spring, Murray C. Killingsworth, Zeljka Calic, Roy G. Beran, Sonu M. M. Bhaskar

**Affiliations:** 1 Global Health Neurology Lab, Sydney, NSW 2000, Australia; 2 Neurovascular Imaging Laboratory, Ingham Institute for Applied Medical Research, Clinical Sciences Stream, Sydney, NSW 2170, Australia; 3 UNSW Medicine and Health, University of New South Wales (UNSW), South West Sydney Clinical Campuses, Sydney, NSW 2170, Australia; 4 NSW Brain Clot Bank, NSW Health Pathology, Sydney, NSW 2170, Australia; 5 Medical Oncology Group, Liverpool Clinical School, Ingham Institute for Applied Medical Research and Western Sydney University (WSU), Sydney, NSW 2751, Australia; 6 School of Medicine, Western Sydney University, Sydney, NSW 2000, Australia; 7 Department of Anatomical Pathology, NSW Health Pathology, Correlative Microscopy Facility, Ingham Institute for Applied Medical Research and Western Sydney University (WSU), Liverpool, NSW 2170, Australia; 8 Department of Neurology & Neurophysiology, Liverpool Hospital & South West Sydney Local Health District (SWSLHD), Sydney, NSW 2170, Australia; 9 Stroke & Neurology Research Group, Ingham Institute for Applied Medical Research, Sydney, NSW 2170, Australia; 10 Griffith Health, School of Medicine and Dentistry, Griffith University, Southport, QLD 4215, Australia

**Keywords:** topography, stroke, reperfusion therapy, ASPECTS, cerebrovascular disorders, neuroimaging

## Abstract

Background: The impact of lesion topography (LT), characterised by the Alberta Stroke Programme Early CT Score (ASPECTS), on outcomes after reperfusion therapy in acute ischemic stroke (AIS) is poorly elucidated. We investigated the prognostic accuracy of ASPECTS-based LT assessment and its association with clinical outcomes in AIS patients considered for reperfusion therapy or receiving intravenous thrombolysis (IVT), endovascular thrombectomy (EVT), or none or both. Methods: Studies were identified from PubMed with additional studies added from Google Scholar. The prevalence of individual ASPECTS regions will also be determined. The association of individual ASPECTS regions with the functional outcome at 90 days will be assessed using random-effects modelling for various cut-offs, such as 6, 7 and 8. The association of continuous ASPECTS with the functional outcome at 90 days will also be undertaken. Forest plots of odds ratios (ORs) will be generated. Results: A total of 25 studies have been included in the final analysis, encompassing 11,404 patients. Pooled estimates indicate that the highest prevalence rates were in cases involving the insula and lentiform nucleus. Subgroup analysis for ASPECTS < 6 (OR 6.10; 95% CI 2.50–14.90; *p* < 0.0001), ASPECTS < 7 (OR 4.58; 95% CI 1.18–17.86; *p* < 0.0001) and ASPECTS < 8 (OR 2.26; 95% CI 1.32–3.89; *p* < 0.0001) revealed a significant association with poor functional outcome at 90 days. Decreasing ASPECTS significantly increased the odds of poor functional outcomes at 90 days (SMD −1.15; 95% CI −1.77–−0.52; *p* < 0.0001). Conclusions: Our meta-analysis demonstrates that decreasing ASPECTS is significantly associated with poor functional outcomes. Individual ASPECTS regions associated with the highest odds of poor functional outcomes were identified. Future studies on the association of LT and clinical outcomes specific to EVT are required.

## 1. Introduction

The advent of reperfusion therapy (RT) has revolutionised the field of stroke medicine [1]. Since 2015, endovascular thrombectomy (EVT) has been incorporated into standards of care, as evidence from six randomised controlled trials (RCTs) on acute ischemic stroke (AIS) caused by large vessel occlusion (LVO) in the extended time window, namely, EXTEND-IA [2], ESCAPE [3], SWIFT-PRIME [4], MR CLEAN [5], REVASCAT [6] and THRACE [7], all of which demonstrated significant clinical benefits and superior reperfusion efficacy. Whether the patient is a good candidate for RT and the appropriateness of specific RT is of clinical relevance and an ongoing research interest. Neuroimaging plays an important role in assessing the lesion topography (LT) or correlates of stroke severity and in acute stroke decision-making in routine clinical practice [8]. The Alberta Stroke Programme Early CT Score (ASPECTS) is a ten-point quantitative topographic score used to determine stroke severity in middle cerebral artery stroke patients. The relevance and clinical utility of LT assessment, using tools such as ASPECTS, and their association with clinical outcomes in AIS patients receiving RT, remain poorly understood and merit further investigation [8,9,10,11].

This study sought to estimate the association of individual ASPECTS regions and ASPECTS cut-off point/s with clinical outcomes in AIS patients considered for RT, or receiving intravenous thrombolysis (IVT), EVT, or none or both, by performing a random-effects meta-analysis. Our underlying questions, in AIS patients considered for, or receiving, RT, are:

1. What is the prevalence of infarcts for each ASPECTS region?
2. What is the average of reported odds ratios for the association of infarcts in each ASPECTS region with functional outcomes at 90 days?
3. What is the reported odds ratio (OR) for the infarcts in each ASPECTS region in left and right hemispheric stroke with functional outcomes at 90 days?
4. Are ASPECTS score cut-offs of 6, 7 and 8 associated with functional outcomes at 90 days?
5. Is there an association of continuous ASPECTS score with functional outcomes at 90 days?

## 2. Methods

### 2.1. Literature Search: Identification and Selection of Studies

**Figure 1:**
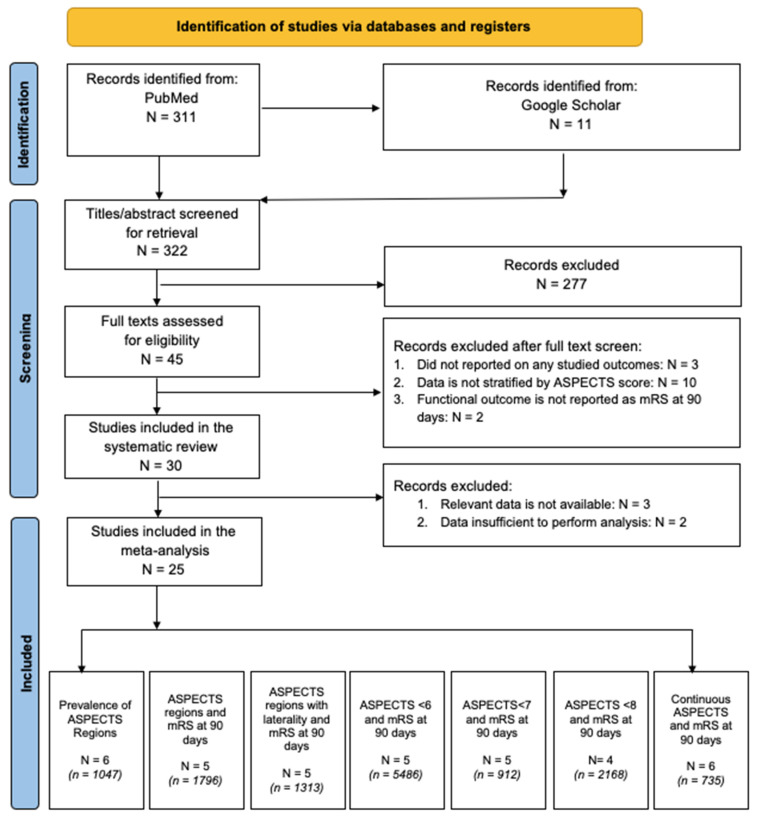
The PRISMA flowchart showing the studies included in the meta-analysis. Abbreviations: N = number of studies; *n* = number of patients; mRS: modified Rankin Score; ASPECTS: Alberta Stroke Programme Early CT Score.

The following databases were searched, PubMed/Medline and Google Scholar, for the period between 1 January 2015 and 10 August 2022. The search terms included (“Stroke” or “brain infarction” or “brain ischemia”) and (“reperfusion” or “thrombectomy” or “endovascular thrombectomy” or “clot retrieval” or “mechanical thrombectomy”) and (“Lesion Topography” or “ASPECTS” or “brain atrophy” or “Hemorrhagic Transformation” or “Intracerebral Hemorrhage” or “Radiological Biomarker” or “Infarct Location” or “Infarct Volume” or “Lesion Volume” or “Laterality” or “Brain Topography”). The full search term/strategy is provided in the Supplementary Materials (Search Strategy). Studies not in the English language and not including human subjects were excluded by applying additional limits. Reference lists of relevant articles, systematic reviews and meta-analyses were also hand-searched to retrieve additional studies. Finally, additional articles were also retrieved from Google Scholar using the keywords/terms specified above. We followed the Preferred Reporting Items for Systematic Reviews and Meta-Analyses (PRISMA) guidelines. This study was registered in Open Science, registration number pg3r8. The PRISMA flowchart shows the search strategy, studies included and various subgroup analyses performed in the meta-analysis (Figure 1). The following reporting frameworks were adhered to: The Meta-analysis of Observational Studies in Epidemiology (MOOSE) checklist (Supplementary Materials Section S2), PRISMA 2020 checklist (Supplementary Materials Section S3) and Standards for Reporting of Diagnostic Accuracy Studies (STARD-2015) checklist (Supplementary Materials Section S4).

### 2.2. Inclusion and Exclusion Criteria

Studies were eligible if they met the following criteria: (a) patients who had experienced AIS who were being considered for RT, including IVT and/or EVT, (b) age ≥ 18 years, (c) hemispheric stroke, (d) availability of data stratified based on ASPECTS score, region, laterality or specific cut-offs, and (e) studies with a good methodological design, with a sufficient sample size, determined to be ≥20 patients in each group. The exclusion criteria were: (1) patients with posterior circulation stroke, (2) animal studies, (3) duplicated publications, (4) full-text articles not available, (5) thrombolytic agent other than tissue plasminogen activator (tPA) used, (6) systematic reviews, meta-analyses, letters and case reports or case series, and (7) studies presented in the abstract form, with relevant data pertaining to ASPECTS (a schematic detailing the ASPECTS is provided in Figure S1) or LT not available or no relevant post-reperfusion clinical outcome measured were excluded. The outcomes measured were: (1) prevalence of infarcts in each ASPECTS region, (2) mean and median estimates of reported ORs for infarcts in each ASPECTS region, (3) mean and median estimates of reported ORs for infarcts in each ASPECTS region with laterality and (4) ORs for poor functional outcomes, defined in terms of the modified Rankin Scale (mRS) 3–6 at 90 days.

### 2.3. Data Extraction

Titles and abstracts, of individual studies retrieved, were reviewed on Endnote, to identify studies mismatched to the eligibility criteria. The remaining articles were thoroughly examined to determine whether they should be included in the systematic review or meta-analysis according to the eligibility criteria. The screening was conducted independently by two authors. Disagreements were discussed and final decisions were reached by consensus. The data from each study/trial were extracted independently using a data extraction sheet to obtain the following information: (1) baseline demographics: title, author and year of publication, (2) study population: age, sample size and characteristics of acute stroke patients, including risk factors, (3) type and time window of the treatments and (4) outcome measures: functional outcome at 90 days measured by the mRS.

### 2.4. Methodological Quality Assessment of Included Studies

The assessment of methodological quality was performed using the Modified Jaded Analysis (MJA) scale, for all studies included in the meta-analysis, independently by two researchers [12]. The risk of bias, owing to funding, was also assessed, based on the declaration of sources of funding and conflicts of interest disclosed in individual studies [13].

### 2.5. Statistical Analysis

All statistical analyses were performed using STATA (Version 13.0, StataCorp LLC, College Station, TX, USA). Baseline characteristics of the overall cohort were derived from individual studies included in the meta-analysis. Mean and standard deviation, as applicable, were calculated from the median and interquartile ranges using the method of Wan et al. [14]. 

The prognostic utility for various ASPECTS cut-off thresholds was evaluated by estimating the pooled sensitivity (SENS) and specificity (SPEC), positive and negative predictive values, positive and negative likelihood ratios and the area under the curve (AUC). AUC is a global measure of prognostic accuracy derived from the summary receiver operating characteristic (SROC) curves. The goodness-of-fit test was performed for each prognostic model. 

To examine the impact of specific ASPECTS cut-off values (6, 7 and 8) and the association of increasing ASPECTS with functional outcomes at 90 days, a random-effects meta-analysis using the DerSimonian and Laird (DL) method was used. Summary effects and heterogeneity measures were tabulated. OR, 95% confidence intervals (CI), percentage weight and heterogeneity across studies were retrieved from the forest plots. I^2^ statistics and *p*-value (<40% = low, 30–60% = moderate, 50–90% = substantial, 75–100% = considerable) were applied to evaluate the heterogeneity between the studies [15]. Subgroup analyses, for different treatment modalities, such as IVT and/or EVT, including the subgroups that did not receive RT despite being considered, were also performed. Egger’s test was used to examine the presence of publication bias. Meta-analysis estimates were also computed using the “meta-inf” command on STATA, to study the influence of individual studies on the overall meta-analysis estimates, when an individual study was excluded. *p*-values < 0.05 were considered as statistically significant.

## 3. Results

### 3.1. Description of Included Studies

A total of 25 studies, comprising 11,404 patients, were included in this meta-analysis. Five studies included patients who primarily received intravenous thrombolysis (IVT), with or without EVT, fourteen studies included patients who primarily received EVT, with or without ST, and six studies include patients who were considered as a candidate of RT but with or without IVT or EVT received. 

Of all patients included in the meta-analysis, 51.9% of patients were male (*n* = 9305), 50.8% of patients had left hemispheric stroke (*n* = 3733) and the mean age ± SD of all included studies was 65.2 ± 14.3 years (*n* = 8720). With regards to overall patients’ clinical characteristics, 66% had hypertension (*n* = 8561), 22.5% had diabetes (*n* = 8565), 34.4% had dyslipidaemia (*n* = 7228), 42.2% had atrial fibrillation (*n* = 7508), 11.9% had prior stroke and/or transient ischaemic attack (TIA) (*n* = 1726) and 20.1% were previous or current smokers (*n* = 7422). See Table 1 for a detailed description of the clinical characteristics of included studies. Table 2 provides summary effects and heterogeneity obtained from the meta-analysis of the association of ASPECTS with clinical outcomes in AIS patients.

Table S1 provides a summary of the level of significance of the association of ASPECTS with the 90-day functional outcome represented by mRS. Subgroup analysis was performed to determine the prognostic capability of ASPECTS in AIS for various reperfusion treatment modalities (Table S2). A publication bias assessment, using Egger’s test of included studies, is summarised in Table S3. The findings of the assessment of methodological quality and funding bias of the included studies are presented in Table S4. Figure S2 provides the findings on the study on the influence of single studies on the overall meta-analysis.

**Table 1:** Clinical characteristics and clinical outcomes of studies included in the meta-analysis.

ID	Authors	Year	Study Type	Cohort	Treatment	Age *	Male	LHS	HTN	Diabetes	Dyslipidaemia	AF	Previous Stroke	Smoking
1	Yu et al. [16]	2021	Prospective	40	No RT ± EVT	60.1 ± 11.8	67.50%		47.50%	37.50%		30.00%		
2	Rangaraju et al. [17]	2015	Retrospective	213	EVT	66.1 ± 14.5		48.83%						
3	Beare et al. [18]	2015	Prospective	185	No RT ± IVT	67.5 ± 12.8	55.14%		70.27%	16.22%				
4	Van Horn et al. [19]	2021	Prospective	123	EVT ± IVT	75.0 ± 14.0	59.35%		65.85%	18.70%	15.45%	37.40%		16.26%
5	Payabvash et al. [20]	2018	Retrospective	198	No RT ± IVT ± EVT	62.7 ± 16.9	62.63%	47.47%	76.77%	29.80%	54.55%	20.71%		41.41%
6	Sheth et al. [15]	2018	Prospective	342	EVT ± IVT	67.0 ± 13.0	42.98%	47.08%	64.91%	16.08%		38.01%	16.08%	16.96%
7	Rosso et al. [21]	2019	Prospective	405	EVT ± IVT	69.7 ± 16.4	54.81%	46.91%						
8	Esmael et al. [22]	2020	Prospective	150	No RT	64.0 ± 11.5	52.67%		68.00%	26.00%	14.67%	18.67%		40.00%
9	Yoo et al. [23]	2016	Prospective	496	IVT ± EVT		58.47%	53.43%	45.36%	13.51%	25.81%	27.22%	10.89%	28.63%
10	Ohta et al. [24]	2018	Retrospective	83	IVT ± EVT	80.6 ± 11.0	48.19%	48.19%	75.90%	22.89%	24.10%	68.67%	16.87%	36.14%
11	Hungerford et al. [25]	2016	Prospective	154	EVT ± IVT	67.2 ± 14.1	50.00%	51.95%						
12	Logan et al. [26]	2018	Prospective	355	EVT ± IVT	67.0 ± 14.0	55.49%		23.10%	1.69%	7.89%	7.61%	3.38%	3.38%
13	Shin et al. [27]	2020	Prospective	350	IVT ± EVT	63.8 ± 12.7	60.29%		56.29%	22.00%		27.71%	13.14%	32.57%
14	Oki et al. [28]	2021	Prospective	688	No RT ± IVT ± EVT	77.0 ± 10.0	52.76%		67.15%	15.70%	29.36%	100.00%		15.99%
15	Ozdemir et al. [29]	2017	Prospective	70	EVT ± IVT	57.0 ± 10.4	58.57%		60.00%	30.00%	58.57%	34.29%		45.71%
16	Wollenweber et al. [30]	2019	Prospective	2637	EVT ± IVT	73.7 ± 13.7	49.62%		75.65%	20.92%	33.86%	40.94%		15.25%
17	Ghodsi et al. [31]	2021	Prospective	553	No RT	65.5 ± 14.4	50.99%		38.52%	66.73%	73.24%			33.27%
18	Seyedsaadat et al. [32]	2021	Prospective	353	EVT ± IVT									
19	Cheng et al. [33]	2021	Retrospective	200	EVT ± IVT	66.2 ± 10.8	58.00%		68.50%	20.50%		41.50%		30.00%
20	Spiotta et al. [34]	2015	Retrospective	149	EVT ± IVT	66.1 ± 15.1	42.95%	53.69%						
21	Jovin et al. [6]	2015	Prospective	206	IVT ± EVT	66.5 ± 10.4	52.91%		65.05%	19.90%			14.56%	
22	Schregel et al. [35]	2018	Prospective	102	EVT	72.8 ± 10.9	47.06%		75.49%	23.53%	42.16%			
23	Deb-Chatterji et al. [36]	2020	Prospective	1700	EVT ± IVT	73.7 ± 13.8	48.94%	50.58%	74.82%	20.92%	32.64%	40.78%		15.4%
24	Kaesmacher et al. [37]	2019	Prospective	1532	EVT ± IVT									
25	Baek et al. [38]	2015	Prospective	120	IVT	66.2 ± 13.2	63.33%		60.83%	22.50%	30.00%	37.5%	18.33%	
Overall				11,404		65.2 ± 14.3 8720/11,404	51.9% 4828/9305	50.58% 1888/3733	66.00% 5650/8561	22.48% 1925/8565	34.37% 2484/7228	42.19% 3168/7508	11.94% 203/1726	20.05% 1488/7422

* Age is demonstrated in the form of mean ± SD. Abbreviations: RT: reperfusion therapy; IVT: intravenous thrombolysis; EVT: endovascular thrombectomy; HTN: hypertension; AF: atrial fibrillation; LHS: left hemispheric stroke. Note: IVT ± EVT: All patients received IVT, with or without EVT. EVT ± IVT: All patients received EVT, with or without IVT. No RT: All patients were considered for reperfusion therapy but were not eligible (received neither IVT nor EVT). No RT ± EVT: All patients were not eligible for IVT, but some received EVT. No RT ± IVT: Some patients received no reperfusion therapy, but a subgroup of patients received IVT. No RT ± IVT ± EVT: Mixed cohort in which some patients received no reperfusion therapy, some received EVT or IVT only, and some received both IVT and EVT.

**Table 2:** Summary effects and heterogeneity obtained from the meta-analysis of the association of ASPECTS with clinical outcomes in acute ischaemic stroke patients considered for, or receiving, reperfusion therapy.

Outcome	Study Groups	Effect Measure	Summary Effects	Heterogeneity ^¶^		Heterogeneity Variance Estimate ^†^		
			Value (95% CI)	Tests of Overall Effect	Cochran’s Q	I^2^ ≤ *	*p*-Value	τ^2^ ≤
Prevalence of infarcts in ASPECTS region	Overall	Prevalence	0.38 (0.33–0.42)	z = 27.91, *p* < 0.001	1945.44	96.97%	*p* < 0.001	8.93
ASPECTS < 6 with poor functional outcome	Overall	OR	6.10 (2.50–14.90)	z = 3.97, *p* < 0.0001	62.01	93.5%	*p* < 0.0001	57.19
	IVT ± EVT		28.99 (15.67–53.61)	z = 10.73, *p* < 0.0001	0.00	N/A	NG ^$^	
	EVT ± IVT		2.69 (2.09–3.46)	z = 7.67, *p* < 0.0001	2.81	28.8%	*p* = 0.246	
	IVT		227.33(13.39–3858.87)	z = 3.76, *p* < 0.0001	0.00	NA	NG ^$^	
ASPECTS < 7 with poor functional outcome	Overall	OR	4.58 (1.18–17.86)	z = 2.19, *p* = 0.028	35.84	88.4%	*p* < 0.0001	35.48
	IVT ± EVT		17.00 (5.08–56.85)	z = 4.60, *p* < 0.0001	0.00	N/A	NG ^$^	
	EVT ± IVT		1.26 (0.79–2.02)	z = 0.97, *p* = 0.330	0.36	0.0%	*p* = 0.835	
	IVT		225.50(28.26–1799.41)	z = 5.11, *p* < 0.0001	0.00	NA	NG ^$^	
ASPECTS < 8 with poor functional outcome	Overall	OR	2.26 (1.32–3.89)	z = 2.95, *p* = 0.003	42.90	81.4%	*p* < 0.0001	1.95
	IVT ± EVT		1.49 (1.02–2.18)	z = 2.06, *p* = 0.040	0.30	0.0%	*p* = 0.582	
	IVT + EVT		1.46 (0.89–2.39)	z = 1.52, *p* = 0.129	0.15	0.0%	*p* = 0.699	
	EVT ± IVT		2.11 (0.78–5.68)	z = 1.47, *p*= 0.140	0.00	NA	NG ^$^	
	IVT		7.25 (0.59–88.54)	z = 1.55, *p* = 0.1.21	34.32	94.2%	*p* < 0.0001	
	No RT		1.61 (1.05–2.47)	z = 2.21, *p* = 0.027	0.00	NA	NG ^$^	
ASPECTS with poor functional outcome	Overall	SMD	−1.15 (−1.77–−0.52)	z = −3.57, *p* < 0.0001	71.97	93.1%	*p* < 0.00001	52.52
	EVT± IVT		−0.77 (−1.60–−0.06)	z = −1.82, *p* = 0.069	12.55	92.0%	*p* < 0.00001	
	EVT		−0.45 (−0.85–−0.05)	z = −2.19, *p* = 0.029	0.00	NA	NG ^$^	
	IVT		−2.70 (−3.21–−2.19)	z = −10.39, *p* < 0.0001	0.00	NA	NG ^$^	
	No RT		−1.52 (−1.90–−1.15)	z = 7.97, *p* < 0.0001	0.00	NA	NG ^$^	
	No RT ± EVT		−0.70 (−1.34–−0.06)	z = −2.14, *p* = 0.032	0.00	NA	NG ^$^	

Abbreviations: ASPECTS: Alberta Stroke Programme Early CT Score; RT: reperfusion therapy; IVT: intravenous thrombolysis; EVT: endovascular thrombectomy; OR: odds ratio; CI: confidence interval; SMD: standardised mean difference; Q: heterogeneity measures were calculated from the data with confidence intervals based on non-central chi-square (common effect) distribution for Cochran’s Q test; H: relative excess in Cochran’s Q over its degrees-of-freedom; I^2^: proportion of total variation in effect estimate due to between-study heterogeneity (based on Cochran’s Q test); τ^2^: among-study variance to test the comparisons of heterogeneity among subgroups; NA: not available. * Values of l≤ are percentages. ^$^ NG: could not be generated. ^¶^ Heterogeneity measures were calculated from the data with 95% confidence intervals based on Gamma (random effects) distribution for Q. ^†^ Heterogeneity variance estimates (tau≤) were derived from the DerSimonian and Laird method. Note: IVT ± EVT: All patients received IVT, with or without EVT. EVT ± IVT: All patients received EVT, with or without IVT. No RT: All patients were considered for reperfusion therapy but were not eligible (received neither IVT nor EVT). No RT ± EVT: All patients were not eligible for IVT, but some received EVT.

### 3.2. Prevalence of ASPECTS Region in AIS Patients

**Figure 2:**
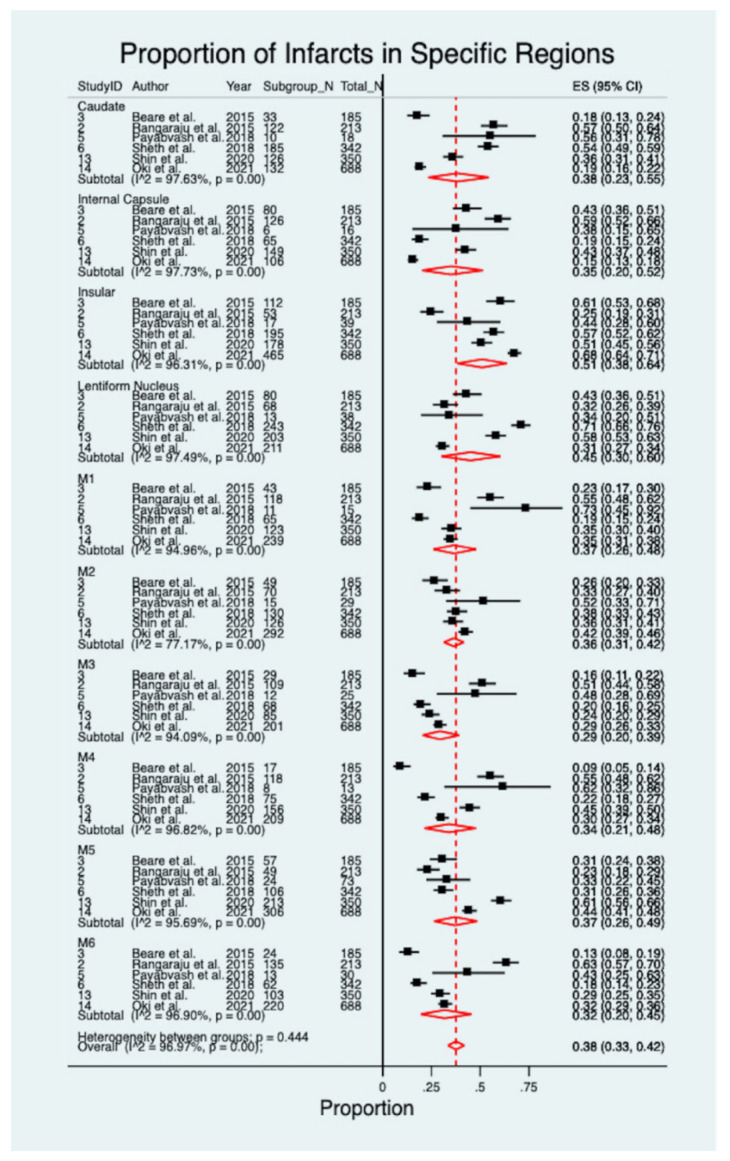
Proportion of infarcts in specific regions [15,17,18,20,27,28].

A meta-analysis of 6 studies, reporting on the prevalence of infarcts in ASPECTS regions for the acute ischemic stroke, encompassing 1047 patients, revealed a significantly high pooled prevalence estimate of 51% of infarcts in the insular region (ES 0.51; 95% 0.38–0.64; z = 11.61, *p* < 0.001), followed by infarcts in the lentiform nucleus (ES 0.45; 95% CI 0.30–0.60; z = 8.86, *p* < 0.001), whereas infarcts in M3 regions have the least pooled estimated prevalence (ES 0.29; 95% CI 0.20–0.39; z = 10.06, *p* < 0.001) (Figure 2) [15,17,18,20,27,28]. Notably, there was moderate to considerable heterogeneity between each ASPECTS region in the included studies (I^2^ = 77.17–97.73%, *p* < 0.001). The estimate of between-study variance (τ^2^) was 8.93 (*p* = 0.44). The crude prevalence for infarcts in any ASPECTS region was 0.37, lower than that of the estimated pooled prevalence of 0.38 (95% CI 0.33–0.42; z = 27.91, *p* < 0.001, I^2^ = 96.97%). The estimated pooled prevalence was lower than, or equal to, the crude prevalence rates observed in each ASPECTS region, expect for infarcts in the caudate, internal capsule, M1, M3, M4 and M6 (Table 3). 

**Table 3:** Pooled estimate of prevalence of infarcts in ten ASPECTS regions.

ASPECTS Region	Study ID	Author	Year	Crude Prevalence	Random Pooled Estimate	95% CI	Significance Test	Heterogeneity Statistic	Heterogeneity *p* Value	I^2^
Caudate	Overall			0.34	0.38	0.23–0.55	z = 7.48, *p* < 0.001	211.00	*p* < 0.001	97.63%
	2	Rangaraju et al. [17]	2015	0.57	0.57	0.50–0.64				
	3	Beare et al. [18]	2015	0.18	0.18	0.13–0.24				
	5	Payabvash et al. [20]	2018	0.56	0.56	0.31–0.78				
	6	Sheth et al. [15]	2018	0.52	0.52	0.47–0.57				
	13	Shin et al. [27]	2020	0.36	0.36	0.31–0.41				
	14	Oki et al. [28]	2021	0.19	0.19	0.16–0.22				
Internal Capsule	Overall			0.30	0.35	0.20–0.52	z = 6.88, *p* < 0.001	219.84	*p* < 0.001	97.73%
	2	Rangaraju et al. [17]	2015	0.59	0.59	0.52–0.66				
	3	Beare et al. [18]	2015	0.43	0.43	0.36–0.51				
	5	Payabvash et al. [20]	2018	0.38	0.38	0.15–0.65				
	6	Sheth et al. [15]	2018	0.19	0.19	0.15–0.24				
	13	Shin et al. [27]	2020	0.43	0.43	0.37–0.48				
	14	Oki et al. [28]	2021	0.15	0.15	0.13–0.18				
Insular	Overall			0.56	0.51	0.38–0.64	z = 11.61, *p* < 0.001	135.49	*p* < 0.001	96.31%
	2	Rangaraju et al. [39]	2015	0.25	0.25	0.19–0.31				
	3	Beare et al. [18]	2015	0.61	0.61	0.53–0.68				
	5	Payabvash et al. [20]	2018	0.44	0.44	0.28–0.60				
	6	Sheth et al. [15]	2018	0.55	0.55	0.50–0.60				
	13	Shin et al. [27]	2020	0.51	0.51	0.45–0.56				
	14	Oki et al. [28]	2021	0.68	0.68	0.64–0.71				
Lentiform Nucleus	Overall			0.45	0.45	0.30–0.60	z = 8.86, *p* < 0.001	198.91	*p* < 0.001	97.49%
	2	Rangaraju et al. [17]	2015	0.32	0.32	0.26–0.39				
	3	Beare et al. [18]	2015	0.43	0.43	0.36–0.51				
	5	Payabvash et al. [20]	2018	0.34	0.34	0.20–0.51				
	6	Sheth et al. [15]	2018	0.70	0.70	0.65–0.75				
	13	Shin et al. [27]	2020	0.58	0.58	0.53–0.63				
	14	Oki et al. [28]	2021	0.31	0.45	0.30–0.60				
M1	Overall			0.33	0.37	0.26–0.48	z = 10.32, *p* < 0.001	99.22	*p* < 0.001	94.96%
	2	Rangaraju et al. [17]	2015	0.55	0.55	0.48–0.62				
	3	Beare et al. [18]	2015	0.23	0.23	0.17–0.30				
	5	Payabvash et al. [20]	2018	0.73	0.73	0.45–0.92				
	6	Sheth et al. [15]	2018	0.19	0.19	0.15–0.24				
	13	Shin et al. [27]	2020	0.35	0.35	0.30–0.30				
	14	Oki et al. [28]	2021	0.35	0.35	0.31–0.38				
M2	Overall			0.38	0.36	0.31–0.42	z = 22.27, *p* < 0.001	21.90	*p* < 0.001	77.17%
	2	Rangaraju et al. [17]	2015	0.33	0.33	0.27–0.40				
	3	Beare et al. [18]	2015	0.26	0.26	0.20–0.33				
	5	Payabvash et al. [20]	2018	0.52	0.52	0.33–0.71				
	6	Sheth et al. [15]	2018	0.38	0.38	0.33–0.43				
	13	Shin et al. [27]	2020	0.36	0.36	0.31–0.41				
	14	Oki et al. [28]	2021	0.42	0.42	0.39–0.46				
M3	Overall			0.28	0.29	0.20–0.39	z = 10.06, *p* < 0.001	84.63	*p* < 0.001	94.09%
	2	Rangaraju et al. [17]	2015	0.51	0.51	0.44–0.58				
	3	Beare et al. [18]	2015	0.16	0.16	0.11–0.22				
	5	Payabvash et al. [20]	2018	0.48	0.48	0.28–0.69				
	6	Sheth et al. [15]	2018	0.20	0.20	0.16–0.25				
	13	Shin et al. [27]	2020	0.24	0.24	0.20–0.29				
	14	Oki et al. [28]	2021	0.15	0.18	0.12–0.27				
M4	Overall			0.33	0.34	0.21–0.48	z = 7.83, *p* < 0.001	157.09	*p* < 0.001	96.82%
	2	Rangaraju et al. [17]	2015	0.55	0.55	0.48–0.62				
	3	Beare et al. [18]	2015	0.09	0.09	0.05–0.14				
	5	Payabvash et al. [20]	2018	0.62	0.62	0.32–0.86				
	6	Sheth et al. [15]	2018	0.22	0.22	0.18–0.27				
	13	Shin et al. [27]	2020	0.45	0.45	0.39–0.50				
	14	Oki et al. [28]	2021	0.30	0.30	0.27–0.34				
M5	Overall			0.41	0.37	0.26–0.49	z = 10.53, *p* < 0.001	115.99	*p* < 0.001	95.69%
	2	Rangaraju et al. [17]	2015	0.23	0.23	0.18–0.29				
	3	Beare et al. [18]	2015	0.31	0.31	0.24–0.38				
	5	Payabvash et al. [20]	2018	0.33	0.33	0.22–0.45				
	6	Sheth et al. [15]	2018	0.30	0.30	0.25–0.35				
	13	Shin et al. [27]	2020	0.61	0.61	0.56–0.66				
	14	Oki et al. [28]	2021	0.44	0.44	0.41–0.48				
M6	Overall			0.31	0.32	0.20–0.45	z = 7.80, *p* < 0.001	161.28	*p* < 0.001	96.90%
	2	Rangaraju et al. [17]	2015	0.63	0.63	0.57–0.70				
	3	Beare et al. [18]	2015	0.13	0.13	0.08–0.19				
	5	Payabvash et al. [20]	2018	0.43	0.43	0.25–0.63				
	6	Sheth et al. [15]	2018	0.28	0.28	0.23–0.33				
	13	Shin et al. [27]	2020	0.29	0.29	0.25–0.35				
	14	Oki et al. [28]	2021	0.32	0.32	0.29–0.36				
Overall ASPECTS Region				0.37	0.38	0.33–0.42	z = 27.91, *p* < 0.001	1945.44	*p* < 0.001	96.97%
Between Subgroups								8.93	*p* = 0.44	

Abbreviations: ASPECTS: Alberta Stroke Programme Early CT Score; CI: confidence interval; I^2^: proportion of total variation in effect estimate due to between-study heterogeneity (based on Cochran’s Q test).

### 3.3. Association of Region-Specific ASPECTS and Laterality with Functional Outcome at 90 Days

Mean and median of reported ORs for poor functional outcome, with infarcts in each ASPECTS region, were estimated from six studies (Table 4) [17,18,27,28,32]. Infarcts in the internal capsule (OR 4.07 ± 6.17) and M3 (OR 4.10 ± 5.70) regions were associated with the highest mean ORs for poor functional outcomes. Infarcts in M1 were associated with the lowest mean ORs for poor functional outcomes (OR 1.84 ± 1.38). When laterality was considered, the analysis of four studies reported different ORs for left and right hemispheric AIS. Infarcts in left M4 (OR 3.15 ± 1.88) and M5 (OR 2.77 ± 0.60) were associated with the highest ORs, whereas infarcts in right M6 (OR 3.79 ± 2.69) and M4 (OR 2.46 ± 1.19) were associated with the highest ORs with poor 90-day functional outcomes (Table 5) [15,17,21,32].

### 3.4. Association of ASPECTS < 6 with Functional Outcome at 90 Days

**Figure 3:**
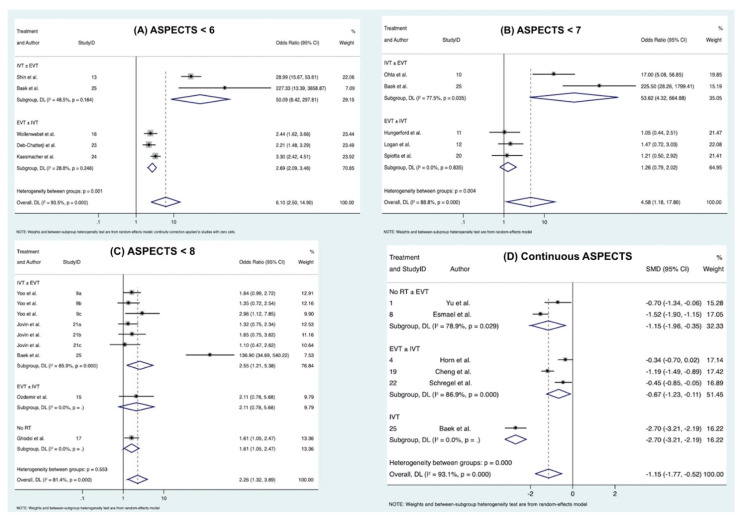
Forest plot of estimated effect for the association of: (**A**) ASPECTS < 6 [27,30,36,37,38], (**B**) ASPECTS < 7 [24,25,26,34,38], (**C**) ASPECTS < 8 [6,23,29,31,38] and (**D**) continuous ASPECTS [16,19,22,33,35,38] with functional outcome at 90 days (mRS 90 days) in acute ischaemic stroke patients receiving, or considered for, reperfusion therapy. Note: IVT ± EVT: All patients received IVT, with or without EVT. EVT ± IVT: All patients received EVT, with or without IVT. No RT: All patients were considered for reperfusion therapy but were not eligible (received neither IVT nor EVT). No RT ± EVT: All patients were not eligible for IVT, but some received EVT. Abbreviations: IVT: intravenous thrombolysis; EVT: endovascular thrombectomy; RT: reperfusion therapy; ASPECTS: Alberta Stroke Programme Early CT Score.

Overall, 5 studies comprising of 5486 patients were included in the meta-analysis of the association of ASPECTS < 6 with poor functional outcome at 90 days [27,30,36,37,38]. ASPECTS less than 6 were significantly associated with increasing odds of poor functional outcome at 90 days (OR 6.10; 95% CI 2.50–14.90; *p* < 0.0001) (Figure 3). Interestingly, the association of ASPECTS < 6 with functional outcome at 90 days was found to be significant in the overall patient cohort, albeit not in both treatment subgroups (Table S1). When stratified by treatment subgroups, associations of ASPECTS < 6 with functional outcome at 90 days in patients receiving EVT, with or without IVT (OR 2.69; 95% CI 2.09–3.46; *p* = 0.246), and in patients receiving IVT, with or without EVT (OR 50.09; 95% CI 8.42–297.81; *p* = 0.154), were not found to be significant. Considerable heterogeneity was found between the studies (I^2^ = 94.4%, *p* < 0.0001). There was evidence of publication bias as revealed by the Egger’s test (Table S3).

### 3.5. Association of ASPECTS < 7 with Functional Outcome at 90 Days

Overall, 5 studies, comprising of 912 patients, were included in the final meta-analysis of the association of ASPECTS < 7 with poor functional outcome at 90 days [24,25,26,34,38]. ASPECTS < 7 was associated with significantly increased odds of poor functional outcomes at 90 days (OR 4.58; 95% CI 1.18–17.86; *p* < 0.0001) (Figure 3). The association of ASPECTS < 7 with functional outcome at 90 days was found to be not significant in patients who received EVT with or without IVT (OR 1.26; 95% CI 0.79–2.02; *p* = 0.835); however, it was significant for the subgroup of patients receiving IVT with or without EVT (OR 53.62; 95% CI 4.32–664.88; *p* = 0.035) (Table S1). Substantial heterogeneity was found between the studies (I^2^ = 88.8%, *p* < 0.0001). There was evidence of publication bias as revealed by the Egger’s test (Table S3). 

### 3.6. Association of ASPECTS < 8 with Functional Outcome at 90 Days

Overall, 4 studies with different treatment subgroups each were included in the meta-analysis of the association of ASPECTS < 8 with poor functional outcome at 90 days, comprising of 2168 patients [6,23,31,38]. ASPECTS < 8 was significantly associated with poor functional outcomes at 90 days (OR 2.26; 95% CI 1.32–3.89; *p* < 0.0001) (Figure 3). Considerable heterogeneity was found between the studies (I^2^ = 81.4%, *p* = 0.825). There was evidence of publication bias as revealed by the Egger’s test (Table S3). Patients receiving IVT with or without EVT had the highest OR with poor functional outcome (OR 2.55; 95% CI 1.21–5.38; *p* < 0.0001), followed by patients receiving EVT with or without IVT (OR 2.11; 95% CI 0.78–5.68) and those receiving no RT (OR 1.6; 95% CI 1.05–2.47). 

### 3.7. Association of Continuous ASPECTS with Functional Outcome at 90 Days

The 6 studies, comprising 735 patients, included in the meta-analysis demonstrated that there was a significant association of increasing ASPECTS with poor functional outcomes at 90 days (SMD = −1.15; 95% CI −1.77–−0.52; *p* < 0.0001) (Figure 3) [16,19,22,33,35,38]. Moderate to considerable heterogeneity was found between the studies (I^2^ = 93.1%, *p* < 0.0001). Patients who received IVT were influenced the most by the decreasing ASPECTS (SMD = −2.70; 95% CI −3.21–−2.19), followed by patients receiving either EVT or no RT (SMD = −1.15; 95% CI −1.96–−0.35; *p* = 0.029), whereas patients receiving EVT, with or without IVT, were least affected (SMD = −0.67; 95% CI −1.23–−0.11; *p* < 0.0001).

## 4. Discussion

Our meta-analysis demonstrates a clear association of LT, as determined using the ASPECTS, with poor functional outcomes in AIS patients being considered for, or receiving, RT. Location of infarcts in the M3 and M6 regions had relatively higher mean ORs for functional dependence at 90 days when compared to other ASPECTS regions. Infarcts in bilateral M4, left M5 and right M6 reported the highest average of reported ORs. Decreasing ASPECTS was significantly associated with poor functional outcome at 90 days when analysed as a continuous variable regardless of the reperfusion treatment modality, with the association being higher for a cut-off score of 6 relative to 8. Different treatment subgroup analyses revealed similar associations with poor functional outcomes; however, associations were not found to be significant in the treatment group of EVT ± IVT pertaining to the ASPECTS < 6 and ASPECTS < 7 subgroups and in the IVT ± EVT group in the ASPECTS < 8 subgroup. 

### 4.1. Infarct Location

Given the increasing use of EVT, as an RT in AIS treatment, using neuroimaging to identify LT associated with clinical profiles in AIS patients receiving RT, it is important to stratify patients for optimal therapy [11]. At a systems level, it warrants a need for specialised pathways to identify AIS patients with a high risk of poor outcomes. It is important to stratify patients’ risks of poor prognosis by their infarct’s location. Our study revealed that infarcts involving the insula are the most prevalent, followed by the involvement of the lentiform nucleus [15,17,18,20,27,28]. This can be attributed to their blood supplies that are mainly from the penetrating arteries, from the M2 segment and the lenticulostriate arteries from M1 segment of middle cerebral artery (MCA), which are both commonly occluded and have poor collateral blood supply [39,40]. Although isolated stroke of the insular or lentiform nucleus are not the most prevalent forms, these two regions are commonly involved when MCA, M1 or M2 are affected and less likely to be reperfused by collateral arteries [41]. Despite being the most affected, these two regions were not the most important in explaining functional outcome at three months, in agreement with previous studies [17,21]. This discrepancy may be explained by the fact that they were rarely infarcted in isolation and the weight of these regions on the functional outcome was lower when other regions were considered.

Previous studies have examined the contribution of individual infarct regions to poor functional outcomes at 90 days, and the reported results show significant disagreement [17,21,42,43]. Some previous studies showed that proximal MCA occlusion is associated with worse outcomes than distal MCA occlusion [42] and the corona radiata, internal capsule and insula have a higher influence on functional outcome at 30 days [43]. Some studies suggested that superficial regions (M1–M6) are associated with worse functional outcomes [17,21], whereas others observed that infarcts in the M1–M3 regions were not associated with poor functional outcomes [27]. Our results suggest that infarcts in all 10 regions had higher means of poor functional outcome at 90 days [17,18,27,28,32]. Infarcts in M3 and internal capsule, which are the two least commonly involved regions, have almost 1.5 times to twice as much influence on functional dependence at 90 days than other ASPECTS regions. This can be attributed to M3 patients having significantly fewer rt-PA and EVT interventions and longer onset-to-hospitalisation times [27].

When laterality was included in the analysis, the results were variable. Two studies demonstrated contradictory results on whether infarcts in M4 and M5 had a protective effect on poor functional outcomes [17,21]. Our study identified that infarcts in left M4 and M5 and in right M6 and M4 were associated with the highest ORs based on the reported ORs from previous studies [15,17,21,32]. We can only speculate on why these reported identified regions are associated with worse outcomes. By composing the superior MCA territory, the M4, M5 and M6 represented the anterior superior frontal lobe, precentral and superior frontal lobe and superior parietal lobe, respectively [10]. Infarcts in the M4, on either side, or M5 might be associated with apraxia, limb weakness and Broca’s dysphasia, which might be expected to have a substantial effect on mRS as it is largely based on mobility. Depending on the dominancy, infarcts in the right M6 will affect either receptive dysphasia or impaired spatial recognition, which affects functional dependence as well. One significant limitation for this analysis is that we only reported on the means and medians of the included studies as the crude ORs could not be calculated due to limited data. The results are prone to selection bias from these studies and may be less reflective of the overall picture.

### 4.2. ASPECTS Value

**Figure 4:**
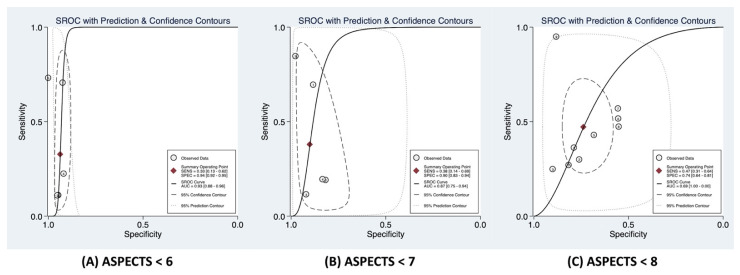
The summary receiver operating characteristics curves for the association of (**A**) ASPECTS < 6, (**B**) ASPECTS < 7 and (**C**) ASPECTS < 8 with functional outcomes at 90 days (mRS 90 days) in acute ischaemic stroke patients considered for, or receiving, reperfusion therapy. Note: the summary receiver operating characteristics curves for the association of continuous ASPECTS with functional outcomes at 90 days (mRS 90 days) in acute ischemic stroke patients receiving reperfusion therapy cannot be generated. Abbreviations: ASPECTS: Alberta Stroke Programme Early CT Score; mRS: modified Rankin Score.

Current evidence on the association of ASPECTS and the functional outcome at 90 days for AIS patients is varied, with discrepant results for varying cut-off values of 6, 7 and 8. Our meta-analysis revealed a clear association of decreasing ASPECTS with odds of poor functional outcomes regardless of the RT received. This is in line with the previous studies that found that decreased ASPECTS are associated with worse functional outcomes [16,19,22,33,35,38]. Despite the study design and cohort size, most of the included studies in this meta-analysis found a significant relationship between poor functional outcomes and ASPECTS cut-off points of 6 [27,30,36,37,38], 7 [24,25,26,34,38] and 8 [6,23,29,31,38]. The ORs with poor functional outcomes doubled as the ASPECTS cut-off decreased from 8 to 7, then increased by approximately 1.3 times when the cut-off further decreased to 6. This also strongly supported our findings about the association between decreased ASPECTS and poor functional outcomes. All three prediction models had relatively high specificity but low sensitivity, which classifies them as predicting tools with a high screening value, but potentially a high false-negative rate (Figure 4). 

Previous studies have examined functional outcome measures such as mortality [31], TICI [29] and sICH [23]. Currently, there are insufficient data to perform a meta-analysis on the comparison of the three cut-off values with these functional outcomes. However, to make a definitive suggestion on which cut-off value is the best to be integrated clinically as a predicting tool to stratify patients for different treatments, further investigation on the association between these other functions is necessary. Factors such as premorbid mRS, previous stroke, stroke laterality, infarct volume and treatment modality need to be taken into consideration individually. It leaves room and direction for future clinical trials and studies in ASPECTS-related prognosis predictions.

### 4.3. Limitations

Our study has several limitations. With regards to the study design, 9 out of 25 studies, included in the meta-analysis, were retrospective, and thus inherently limited in their design. This resulted in a significant proportion of the included studies relying on stroke centres and clinicians to decide on the treatment, which leads to difficulties in selecting a specific reperfusion therapy modality as the intervention group, and patients who received different types of RT were mixed in the overall study cohort. In terms of the reporting of patient characteristics, certain parameters, such as premorbid status, ASPECTS, National Institutes for Health Stroke Scale (NIHSS) and the number of patients who received which type of RT, were either minimally reported or not reported at all throughout all the included studies. Studies on the association of ASPECTS with other prognostic parameters, such as mortality, post-procedural recanalization, sICH and hypertension (HT), could not be performed due to the limited data from large, randomised controlled trials in the new and understudied field of EVT in stroke medicine. Lastly, our analyses of ASPECTS < 7 and ASPECTS < 8 with the poor functional outcome at 90 days had relatively small cohorts when compared to that of ASPECS < 6 and were not highly powered for horizontal comparison. Our findings should be interpreted in the context of the methodological design and the study population. Given that we performed a random-effects model, some of these variabilities and heterogeneities presumably should be accounted for. 

## 5. Conclusions

ASPECTS location and specific ASPECTS thresholds are important clinical considerations for evaluation and prognostication in AIS patients considered for, or receiving, RT. Our meta-analysis clearly demonstrates that LT, determined using ASPECTS, is associated with poor functional outcomes at 90 days, and these findings are mostly consistent across reperfusion treatment subgroups. These findings indicate the prognostic utility of LT assessment, using tools such as ASPECTS, in AIS patients considered for, or receiving, RT. Future studies on the optimal threshold of ASPECTS as well as the role of laterality in acute stroke prognostication are warranted.

## Figures and Tables

**Table 4:** Summary of mean and median of reported odds ratios for infarcts in 10 ASPECTS regions with functional outcomes at 90 days.

ASPECTS Region	No. of Studies Included	Mean OR * ± SD	Median OR * (IQR)
Caudate	5	1.97 ± 0.78	1.74 (1.50–2.09)
Internal Capsule	6	4.07 ± 6.17	1.72 (1.03–2.58)
Insular	6	2.00 ± 0.90	1.66 (1.36–2.46)
Lentiform Nucleus	6	1.90 ± 1.05	1.42 (1.22–2.86)
M1	5	1.84 ± 1.38	1.75 (1.07–1.94)
M2	5	3.00 ± 2.00	1.90 (1.55–4.19)
M3	5	4.10 ± 5.70	1.33 (0.86–3.50)
M4	6	2.38 ± 0.91	2.80 (1.66–2.94)
M5	6	2.56 ± 0.97	1.84 (1.51–3.10)
M6	6	2.98 ± 1.68	2.68 (1.67–3.00)

* Crude ORs cannot be calculated due to limited data available. Abbreviations: ASPECTS: Alberta Stroke Programme Early CT Score; SD: standard deviation; IQR: inter-quartile range.

**Table 5:** Summary of mean and median of ORs for infarcts in 10 ASPECTS regions, stratified by laterality, with functional outcomes at 90 days.

ASPECTS Region	No. of Studies	Left		Right	
		Mean OR * ± SD	Median OR * (IQR)	Mean OR * ± SD	Median OR * (IQR)
Caudate	4	1.67 ± 1.43	1.72 (0.50–2.84)	1.84 ± 1.25	2.37 (0.42–2.74)
Internal Capsule	4	2.23 ± 2.61	1.14 (0.80–3.66)	1.12 ± 0.78	1.32 (0.63–1.62)
Insular	4	1.49 ± 1.06	1.71 (0.84−2.14)	1.49 ± 1.30	1.41 (0.57–2.40)
Lentiform Nucleus	4	1.49 ± 1.12	1.72 (0.64–2.35)	0.57 ± 0.46	0.81 (0.04–0.87)
M1	4	1.65 ± 1.13	1.44 (0.79–2.50)	1.41 ± 1.27	1.09 (0.64–2.18)
M2	4	1.68 ± 0.51	1.76 (1.34–2.02)	2.07 ± 1.40	2.27 (1.09–3.04)
M3	4	1.93 ± 1.21	1.98 (0.91–2.96)	1.68 ± 2.23	0.75 (0.36–3.01)
M4	4	3.15 ± 1.88	2.95 (1.98–4.33)	2.46 ± 1.19	2.62 (1.59–3.34)
M5	4	2.77 ± 0.60	2.73 (2.37–3.17)	1.46 ± 1.00	1.69 (0.73–2.19)
M6	4	1.48 ± 1.17	1.00 (0.84–2.12)	3.79 ± 2.69	4.43 (1.89–5.69)

* Crude ORs cannot be calculated due to limited data available. Abbreviations: OR: odds ratios; ASPECTS: Alberta Stroke Programme Early CT Score; SD: standard deviation; IQR: inter-quartile range.

## Data Availability

The original contributions presented in the study are included in the article/Supplementary Materials, and further inquiries can be directed to the corresponding author.

## Supplementary Materials

The following supporting information can be downloaded online at: https://www.mdpi.com/article/10.3390/neurolint14040073/s1. Figure S1: Schematic of ASPECTS; Figure S2: Influence of single studies on the overall meta-analysis; Table S1: Summary of significant meta-analysis outcomes; Table S2: Summary data and performance estimates for selectivity and specificity analysis; Table S3: Egger’s test for publication bias assessment of the included studies; Table S4: Jaded analysis for methodological quality/risk of bias and test for funding bias. References [6,15,16,17,18,19,20,21,22,23,24,25,26,27,28,29,30,31,32,33,34,35,36,37,38,44] are cited in the supplementary materials.
