# Supplementary material for: Association of Lesion Topography with Functional Outcomes in Acute Ischemic Stroke Patients Considered for, or Receiving, Reperfusion Therapy: A Meta-Analysis
Source: Neurol Int. 2022 Nov 7;14(4):903–22. doi: 10.3390/neurolint14040073 (PMC9680454; doi:10.3390/neurolint14040073)
Supplement: Supplementary file 1 [file neurolint-14-00073-s001.zip › neurolint-1958092-supplementary.pdf]

Supplementary Materials

Association of Lesion Topography with Functional Outcomes in Acute Ischemic Stroke Patients Receiving Reperfusion Therapy: A Meta-Analysis

Shuyue Chen, Kevin J. Spring, Murray C. Killingsworth, Zeljka Calic, Roy G. Beran and Sonu M. M. Bhaskar

List of Contents

1. Search Strategy (Keywords/MeSH Terms)
2. MOOSE Checklist for meta-analyses of observational studies included in the study
3. PRISMA checklist for the meta-analysis
4. STARD-2015 Checklist for prognostic studies
5. Supplemental Tables:
   5A. Table S1. Summary of significant meta-analysis outcomes
   5B. Table S2. Summary data and performance estimates for selectivity and specificity analysis
   5C. Table S3. Egger's test for publication bias assessment of the included studies
   5D. Table S4. Jaded analysis for methodological quality/risk of bias and test for funding bias
6. Supplemental Figures:
   6A. Figure S1. Schematic of ASPECTS
   6B. Figure S2. Influence of single studies on the overall meta-analysis

S1. Search Strategy (Keywords/MeSH Terms)

("Stroke" OR "brain infarction" OR "brain ischemia") AND ("reperfusion" OR "thrombectomy" OR "endovascular thrombectomy" OR "clot retrieval" OR "mechanical thrombectomy") AND ("Lesion Topography" OR "ASPECTS" OR "brain atrophy" OR "Hemorrhagic Transformation" OR "Intracerebral Hemorrhage" OR "Radiological Biomarker" OR "Infarct Topography" OR "Infarct Location" OR "Infarct Volume" OR "Lesion Volume" OR "Laterality" OR "Brain Topography").

S2. MOOSE Checklist for meta-analyses of observational studies included in the study

Item No.	Recommendation	Reported on Page No.
	**Reporting of background should include**	
1	Problem definition	7
2	Hypothesis statement	7
3	Description of study outcome(s)	8
4	Type of exposure or intervention used	8
5	Type of study designs used	-
6	Study population	8
	**Reporting of search strategy should include**	
7	Qualifications of searchers (e.g., librarians and investigators)	1
8	Search strategy, including time period included in the synthesis and key words	7, Supplemental Table 1
9	Effort to include all available studies, including contact with authors	-
10	Databases and registries searched	7
11	Search software used, name and version, including special features used (e.g., explosion)	9
12	Use of hand searching (e.g., reference lists of obtained articles)	7
13	List of citations located and those excluded, including justification	8–9, Table 1
14	Method of addressing articles published in languages other than English	-
15	Method of handling abstracts and unpublished studies	-
16	Description of any contact with authors	-
	**Reporting of methods should include**	
17	Description of relevance or appropriateness of studies assembled for assessing the hypothesis to be tested	7
18	Rationale for the selection and coding of data (e.g., sound clinical principles or convenience)	-
19	Documentation of how data were classified and coded (e.g., multiple raters, blinding and inter-rater reliability)	-
20	Assessment of confounding (e.g., comparability of cases and controls in studies where appropriate)	-
21	Assessment of study quality, including blinding of quality assessors, stratification, or regression on possible predictors of study results	6, Supplemental Table 4
22	Assessment of heterogeneity	9–10, Table 2
23	Description of statistical methods (e.g., complete description of fixed or random effects models, justification of whether the chosen models account for predictors of study results, dose-response models, or cumulative meta-analysis) in sufficient detail to be replicated	9–10
24	Provision of appropriate tables and graphics	List of tables/figures, Supplemental information
	**Reporting of results should include**	
25	Graphic summarising individual study estimates and overall estimate	-
26	Table giving descriptive information for each study included	21–22 (Table1)
27	Results of sensitivity testing (e.g., subgroup analysis)	9, Supplemental Table 2
28	Indication of statistical uncertainty of findings	-

S3. PRISMA Checklist for the meta-analysis [44]

Section and Topic	Item #	Checklist item	Location where item is reported
		**TITLE**	
Title	1	Identify the report as a systematic review.	N/A
		**ABSTRACT**	
Abstract	2	See the PRISMA 2020 for Abstracts checklist.	6
		**INTRODUCTION**	
Rationale	3	Describe the rationale for the review in the context of existing knowledge.	7
Objectives	4	Provide an explicit statement of the objective(s) or question(s) the review addresses.	7
		**METHODS**	

Eligibility criteria	5	Specify the inclusion and exclusion criteria for the review and how studies were grouped for the syntheses.	8–9
Information sources	6	Specify all databases, registers, websites, organisations, reference lists and other sources searched or consulted to identify studies. Specify the date when each source was last searched or consulted.	8–9
Search strategy	7	Present the full search strategies for all databases, registers and websites, including any filters and limits used.	Supplemental information 1
Selection process	8	Specify the methods used to decide whether a study met the inclusion criteria of the review, including how many reviewers screened each record and each report retrieved, whether they worked independently, and if applicable, details of automation tools used in the process.	8–9
Data collection process	9	Specify the methods used to collect data from reports, including how many reviewers collected data from each report, whether they worked independently, any processes for obtaining or confirming data from study investigators, and if applicable, details of automation tools used in the process.	8–9
Data items	10a	List and define all outcomes for which data were sought. Specify whether all results that were compatible with each outcome domain in each study were sought (e.g., for all measures, time points, analyses), and if not, the methods used to decide which results to collect.	8–9
	10b	List and define all other variables for which data were sought (e.g., participant and intervention characteristics, funding sources). Describe any assumptions made about any missing or unclear information.	8–9
Study risk of bias assessment	11	Specify the methods used to assess risk of bias in the included studies, including details of the tool(s) used, how many reviewers assessed each study and whether they worked independently, and if applicable, details of automation tools used in the process.	9
Effect measures	12	Specify for each outcome the effect measure(s) (e.g., risk ratio, mean difference) used in the synthesis or presentation of results.	8–9
Synthesis methods	13a	Describe the processes used to decide which studies were eligible for each synthesis (e.g., tabulating the study intervention characteristics and comparing against the planned groups for each synthesis (item #5)).	-
	13b	Describe any methods required to prepare the data for presentation or synthesis, such as handling of missing summary statistics, or data conversions.	9
	13c	Describe any methods used to tabulate or visually display results of individual studies and syntheses.	9
	13d	Describe any methods used to synthesise results and provide a rationale for the choice(s). If meta-analysis was performed, describe the model(s)/method(s) to identify the presence and extent of statistical heterogeneity, and software package(s) used.	9
	13e	Describe any methods used to explore possible causes of heterogeneity among study results (e.g., subgroup analysis, meta-regression).	10
	13f	Describe any sensitivity analyses conducted to assess robustness of the synthesised results.	10
Reporting bias assessment	14	Describe any methods used to assess risk of bias due to missing results in a synthesis (arising from reporting biases).	-
Certainty assessment	15	Describe any methods used to assess certainty (or confidence) in the body of evidence for an outcome.	10
**RESULTS**			
Study selection	16a	Describe the results of the search and selection process, from the number of records identified in the search to the number of studies included in the review, ideally using a flow diagram.	Supplemental information 1
	16b	Cite studies that might appear to meet the inclusion criteria, but which were excluded, and explain why they were excluded.	-
Study characteristics	17	Cite each included study and present its characteristics.	Table 1, Reference
Risk of bias in studies	18	Present assessments of risk of bias for each included study.	Supplemental information

Results of individual studies	19	For all outcomes, present, for each study: (a) summary statistics for each group (where appropriate) and (b) an effect estimates and its precision (e.g., confidence/credible interval), ideally using structured tables or plots.	8–9
Results of syntheses	20a	For each synthesis, briefly summarise the characteristics and risk of bias among contributing studies.	10–13
	20b	Present results of all statistical syntheses conducted. If meta-analysis was done, present for each the summary estimate and its precision (e.g., confidence/credible interval) and measures of statistical heterogeneity. If comparing groups, describe the direction of the effect.	10–13
	20c	Present results of all investigations of possible causes of heterogeneity among study results.	-
	20d	Present results of all sensitivity analyses conducted to assess the robustness of the synthesised results.	Table 2
Reporting biases	21	Present assessments of risk of bias due to missing results (arising from reporting biases) for each synthesis assessed.	10–13
Certainty of evidence	22	Present assessments of certainty (or confidence) in the body of evidence for each outcome assessed.	10–13
**DISCUSSION**			
Discussion	23a	Provide a general interpretation of the results in the context of other evidence.	13–14
	23b	Discuss any limitations of the evidence included in the review.	13–16
	23c	Discuss any limitations of the review processes used.	13–16
	23d	Discuss implications of the results for practice, policy and future research.	15–16
**OTHER INFORMATION**			
Registration and protocol	24a	Provide registration information for the review, including register name and registration number, or state that the review was not registered.	-
	24b	Indicate where the review protocol can be accessed, or state that a protocol was not prepared.	-
	24c	Describe and explain any amendments to information provided at registration or in the protocol.	-
Support	25	Describe sources of financial or non-financial support for the review, and the role of the funders or sponsors in the review.	13
Competing interests	26	Declare any competing interests of review authors.	13
Availability of data, code and other materials	27	Report which of the following are publicly available and where they can be found: template data collection forms, data extracted from included studies, data used for all analyses, analytic code and any other materials used in the review.	13

S4. STARD-2015 Checklist for prognostic studies

Section and Topic	No.	Item	Reported on page #
**TITLE OR ABSTRACT**			
	1	Identification as a study of diagnostic accuracy using at least one measure of accuracy<br>(such as sensitivity, specificity, predictive values, or AUC)	9–10
**ABSTRACT**			
	2	Structured summary of study design, methods, results and conclusions<br>(for specific guidance, see STARD for Abstracts)	6
**INTRODUCTION**			
	3	Scientific and clinical background, including the intended use and clinical role of the index test	6–7
	4	Study objectives and hypotheses	7
**METHODS**			
Study design	5	Whether data collection was planned before the index test and reference standard were performed (prospective study) or after (retrospective study)	-
Participants	6	Eligibility criteria	8–9

	7	On what basis potentially eligible participants were identified (such as symptoms, results from previous tests, inclusion in registry)	8–9
	8	Where and when potentially eligible participants were identified (setting, location and dates)	8–9
	9	Whether participants formed a consecutive, random or convenience series	-
Test methods	10a	Index test, in sufficient detail to allow replication	-
	10b	Reference standard, in sufficient detail to allow replication	-
	11	Rationale for choosing the reference standard (if alternatives exist)	-
	12a	Definition of and rationale for test positivity cut-offs or result categories of the index test, distinguishing pre-specified from exploratory	10
	12b	Definition of and rationale for test positivity cut-offs or result categories of the reference standard, distinguishing pre-specified from exploratory	-
	13a	Whether clinical information and reference standard results were available to the performers/readers of the index test	-
	13b	Whether clinical information and index test results were available to the assessors of the reference standard	-
Analysis	14	Methods for estimating or comparing measures of diagnostic accuracy	9–10
	15	How indeterminate index test or reference standard results were handled	-
	16	How missing data on the index test and reference standard were handled	-
	17	Any analyses of variability in diagnostic accuracy, distinguishing pre-specified from exploratory	-
	18	Intended sample size and how it was determined	-
**RESULTS**			
Participants	19	Flow of participants, using a diagram	Figure 1
	20	Baseline demographic and clinical characteristics of participants	10, Table 1
	21a	Distribution of severity of disease in those with the target condition	-
	21b	Distribution of alternative diagnoses in those without the target condition	-
	22	Time interval and any clinical interventions between index test and reference standard	-
Test results	23	Cross-tabulation of the index test results (or their distribution) by the results of the reference standard	Table 1
	24	Estimates of diagnostic accuracy and their precision (such as 95% confidence intervals)	-
	25	Any adverse events from performing the index test or the reference standard	-
**DISCUSSION**			
	26	Study limitations, including sources of potential bias, statistical uncertainty and generalisability	13–16
	27	Implications for practice, including the intended use and clinical role of the index test	13–16
**OTHER INFORMATION**			
	28	Registration number and name of registry	-
	29	Where the full study protocol can be accessed	Main Manuscript and Supplemental Information
	30	Sources of funding and other support, role of funders	13

S5. Supplemental Tables

Table S1. Summary of significant meta-analysis outcomes.

	**Prevalence of infarcts in ASPECTS region**	**ASPECTS < 6 with functional outcome at 90 days**	**ASPECTS < 7 with functional outcome at 90 days**	**ASPECTS < 8 with functional outcome at 90 days**	**ASPECTS with functional outcome at 90 days**
Overall	Significant	Significant	Significant	Significant	Significant
EVT ± IVT	NA	Not significant	Not significant	Significant	Significant
IVT ± EVT	NA	Not significant	Significant	Not significant	NA

IVT	NA	NA	NA	NA	Significant
No RT	NA	NA	NA	Significant	NA
No RT ± EVT	NA	NA	NA	NA	Significant

*NA: Data are not available for this treatment subgroup.

Table S2. Summary data and performance estimates for selectivity and specificity analysis.

	**ASPECTS < 6 with mRS at 90 days, *n* (95% CI)**	**ASPECTS < 7 with mRS at 90 days, *n* (95% CI)**	**ASPECTS < 8 with mRS at 90 days, *n* (95% CI)**	**ASPECTS with mRS at 90 days, *n* (95% CI)**
Sensitivity	0.33 (0.13–0.62)	0.38 (0.14–0.69)	0.47 (0.31–0.64)	0.48 (0.31–0.65)
Specificity	0.94 (0.92–0.95)	0.90 (0.83–0.94)	0.74 (0.64–0.81)	0.65 (0.49–0.79)
ROC Area, AUROC	0.93 (0.88–0.96)	0.87 (0.75–0.94)	0.69 (1.00–0.00)	0.58 (0.46–0.71)
PLR	5.2 (2.4–11.4)	3.8 (1.2–12.6)	1.8 (1.1–3.0)	1.4 (1.1–1.6)
NLR	0.72 (0.49–1.06)	0.69 (0.41–1.17)	0.72 (0.50–1.02)	0.8 (0.68–0.94)
Prognostic Odds Ratio	7 (2–23)	6 (1–30)	2 (1–6)	2 (1–2)
AIC	100.8	78.6	157	85.1
BIC	102.3	80.2	161.5	86.6
Deviance	90.8	68.6	147	75.1
BICdiff	413.3	152.7	151.6	122.0
Correlation (Mixed Model)	−0.53	0.69	0.02	−1.00
Proportion of heterogeneity likely due to threshold effect	0.28	0.47	0.00	1.00
ICC_SEN	0.36 (0.07–0.65)	0.40 (0.09–0.70)	0.25 (0.05–0.45)	0.15 (0.00–0.33)
MED_SEN	0.79 (0.67–0.92)	0.80 (0.68–0.94)	0.73 (0.64–0.84)	0.67 (0.59–0.82)
ICC_SPE	0.01 (0.00–0.03)	0.08 (0.00–0.24)	0.10 (0.00–0.22)	0.15 (0.00–0.31)
MED_SPE	0.54 (0.51–0.65)	0.62 (0.54–0.84)	0.64 (0.58–0.75)	0.67 (0.59–0.80)
Heterogeneity (Chi-square):	LRT_Q = 78.468, df = 2.00, LRT_p = 0.000	LRT_Q = 15.289, df = 2.00, LRT_p = 0.000	LRT_Q = 69.635, df = 2.00, LRT_p = 0.000	LRT_Q = 110.911, df =2.00, LRT_p = 0.000
Inconsistency (I^2)	LRT_I^2 = 97% CI = (96–99)	LRT_I^2 = 87% CI = (73–100)	LRT_I^2 = 97% CI = (95–99)	LRT_I^2= 98, 95% CI = (97–99)

Table S3. Egger's test for publication bias assessment of the included studies.

**Outcome**	**Std. Eff.**	**Coef.**	**Std. Err.**	**t**	**P > [t]**	**95% CI**	***p*-value**
**ASPECTS < 6 mRS**	Slope	0.20	0.81	0.25	0.821	−2.39–2.79	0.241
	Bias	5.24	3.59	1.46	0.241	−6.19–16.66	
**ASPECTS < 7 mRS**	Slope	−3.04	1.00	−3.02	0.057	−6.24–0.17	0.028
	Bias	8.19	2.06	3.98	0.028	1.64–14.75	
**ASPECTS < 8 mRS**	Slope	−0.73	0.68	−1.08	0.318	−2.32–0.87	0.082
	Bias	4.05	2.00	2.03	0.082	−0.68–8.78	

Table S4. Jaded analysis for methodological quality/risk or bias and test for funding bias [6,15–38].

Jaded Analysis	Yu et al.	Rangaraju et al.	Beare et al.	Horn et al.	Payabvash et al.	Sheth et al.	Rosso et al.	Esmael et al.	Yoo et al.	Ohta et al.	Hungerford et al.	Logan et al.	Shin et al.
Was the study randomised?	0	0	0	0	0	0	0	0	0	0	0	0	0
Was the method of randomisation appropriate? (not specified = 0)	0	0	0	0	0	0	0	0	0	0	0	0	0
Was the study described as being blinded?	0	0	0	0	0	1	1	0	0	0	1	0	0
Was the method of blinding appropriate?	0	0	0	0	0	1	1	0	0	0	1	0	0
Was there a description of withdrawals and dropouts?	0	0	0	0	0	0	0	0	0	0	0	0	0
Was there a clear description of the inclusion/exclusion criteria?	1	1	1	1	1	1	1	1	1	1	1	1	1
Was the method used to assess adverse events described?	0	0	0	0	0	0	0	0	0	0	0	0	0
Was the method of statistical analysis described?	1	1	1	1	1	1	1	1	1	1	1	1	1
**TOTAL (MJA_ROB)**	**2**	**2**	**2**	**2**	**2**	**4**	**4**	**2**	**2**	**2**	**2**	**2**	**2**
Funding bias	0	0	0	0	0	2	2	0	1	0	1	0	0

Jaded Analysis	Oki et al.	Ozdemir et al.	Wollenweber et al.	Ghodsi et al.	Seyedsaadat et al.	Cheng et al.	Spiotta et al.	Jovin et al.	Schregel et al.	Deb-Chatterji et al.	Kaesmacher et al.	Baek et al.
Was the study randomised?	0	0	0	0	0	0	0	1	0	0	0	0
Was the method of randomisation appropriate? (not specified = 0)	0	0	0	0	0	0	0	1	0	0	0	0
Was the study described as being blinded?	0	0	0	1	1	1	1	1	1	0	0	1
Was the method of blinding appropriate?	0	0	0	1	1	1	1	1	1	0	0	1
Was there a description of withdrawals and dropouts?	1	0	0	0	0	0	0	1	0	0	0	0
Was there a clear description of the inclusion/exclusion criteria?	1	1	1	1	1	1	1	1	1	1	1	1
Was the method used to assess adverse events described?	0	0	0	0	0	0	0	1	0	0	0	0
Was the method of statistical analysis described?	1	1	1	1	1	1	1	1	1	1	1	1
**TOTAL (MJA_ROB)**	3	2	2	4	4	4	4	8	4	2	2	4
Funding bias	0	0	0	0	0	0	0	?	0	0	0	0

S6. Supplemental Figures

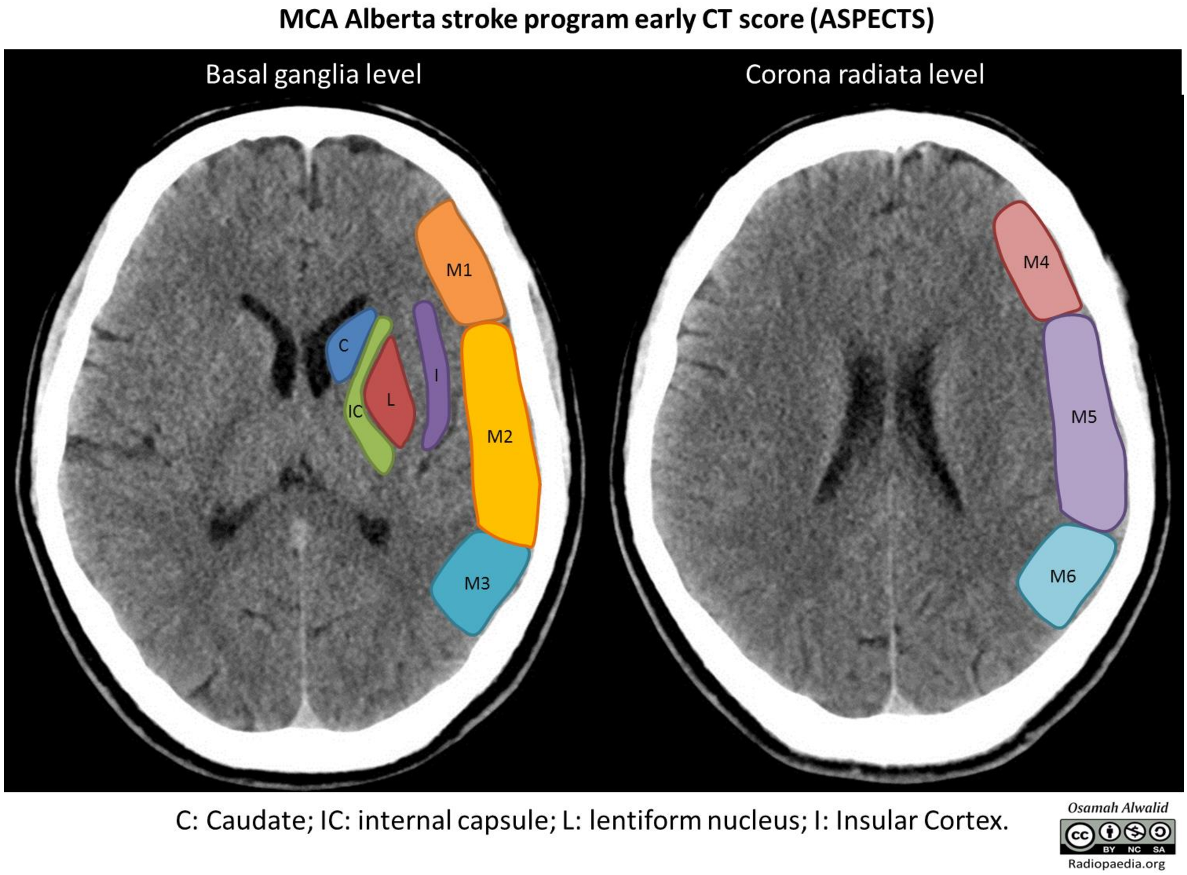

Figure S1. Schematic of ASPECTS. Note: Case courtesy of Dr Henry Knipe, Dr Haris Sair, and Dr Osamah A. A. Alwalid, Radiopaedia.org, rID: 72706. From the case https://radiopaedia.org/articles/alberta-stroke-programme-early-ct-score-aspects (accessed on 31 Oct 2022).

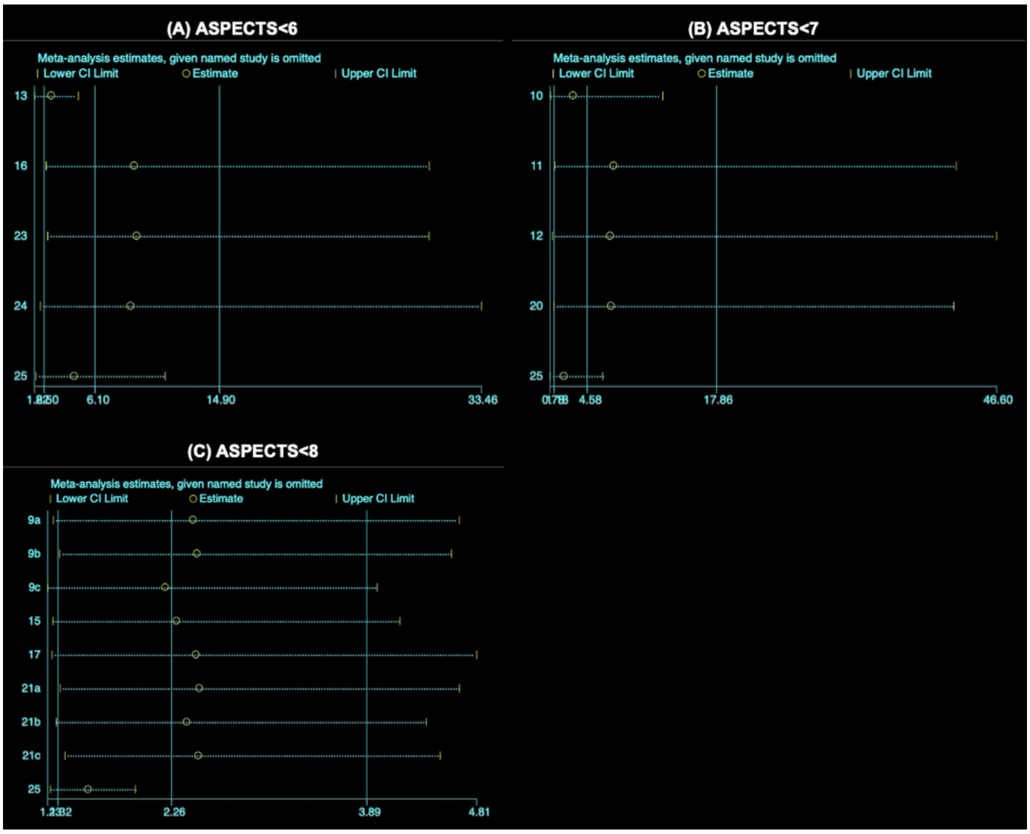

Figure S2. Influence of single studies on the overall meta-analysis. Note: the influence of single studies on the overall meta-analysis graph for subgroup analysis of the association of continuous ASPECTS with functional outcome at 90 days (mRS 90 days) could not be generated.
